# Association between metabolic syndrome and prognosis of breast cancer: a meta-analysis of follow-up studies

**DOI:** 10.1186/s13098-019-0514-y

**Published:** 2020-01-29

**Authors:** Peiting Li, Tianying Wang, Chen Zeng, Meng Yang, Gang Li, Jiang Han, Wei Wu

**Affiliations:** 1 Department of Breast Thyroid Surgery, The Third Xiangya Hospital of Central South University, No. 172 Tong Zi Po Road, Changsha, 410013 China; 2 Department of Hepatopancreatobiliary Surgery, The Third Xiangya Hospital of Central South University, No. 172 Tong Zi Po Road, Changsha, 410013 China

**Keywords:** Metabolic syndrome, Breast cancer, Recurrence, Mortality, Meta-analysis

## Abstract

**Background:**

Metabolic syndrome (MetS) has been suggested to be a risk factor for many cancers, including breast cancer. However, it remains unclear whether MetS predicts poor prognosis in women with breast cancer. A meta-analysis was performed to summarize the association between MetS and clinical outcome in women with breast cancer.

**Methods:**

Cohort studies were identified by search of PubMed and Embase databases. A random-effect model incorporating the potential heterogeneity was applied to pool the results. Subgroup analyses according to the ethnicity and study design were performed.

**Results:**

Nine cohort studies with 17,892 women with breast cancer were included. Pooled results showed that MetS was significantly associated with an increased risk of breast cancer recurrence (adjusted risk ratio [RR] = 1.52, 95%, p = 0.02). Subgroup analyses showed that MetS was independently associated with increased recurrence of breast cancer in Caucasians (adjusted RR = 1.75, p = 0.02), but not in Asians (adjusted RR = 1.07, p = 0.81), and MetS was associated with a trend of increased risk of breast cancer recurrence in both the prospective and retrospective studies. Although we failed to show a significant association between MetS and breast cancer related deaths (adjusted RR = 1.24, p = 0.41), MetS was associated with increased risk of all-cause deaths in these patients (adjusted RR = 1.80, p < 0.001).

**Conclusions:**

MetS may predict the risk of cancer recurrence and mortality in women with breast cancer, particularly in Caucasians.

## Background

Despite of the improvements in the prevention and management of cancer, breast cancer remains a common malignancy in women, and about 1.4 million women are diagnosed with breast cancer annually [1–3]. Metabolic disorders, such as obesity and insulin resistance, have been suggested to be involved in the pathogenesis and progression of breast cancer [4–6]. Metabolic syndrome (MetS), which indicates a cluster of metabolic abnormalities including abdominal adiposity, insulin resistance, hyperglycemia, hypertension, and dyslipidemia [7–10], has been considered to be a risk factor of a variety of cancers, including breast cancer in postmenopausal women [11, 12]. In addition, MetS have been proposed as a prognostic factor in women with breast cancer. Particularly, MetS has been associated with more aggressive tumor biology of breast cancer [13, 14], and some studies showed that MetS is associated with higher risk of recurrence and mortality in these patients [15–18]. However, other cohort studies failed to show a significant association between MetS and poor clinical outcomes in women with breast cancer [19–23]. Moreover, whether factors such as ethnicity and study design affects the association between MetS and prognosis in women with breast cancer remains to be determined [24]. Therefore, in this study, we performed a meta-analysis to evaluate the potential association between MetS and risks of recurrence or death in women with breast cancer.

## Methods

The meta-analysis was designed and performed in accordance with the MOOSE (Meta-analysis of Observational Studies in Epidemiology) [25] and Cochrane’s Handbook [26] guidelines.

### Literature search

Electronic databases of PubMed and Embase were systematically searched using the combination of the following terms: (1) “metabolic syndrome” OR “insulin resistance syndrome” OR “syndrome X”; (2) “breast cancer”; and (3) “survival” OR “prognosis” OR “mortality” OR “death” OR “recurrence” OR “surgery” OR “operation”. The search was limited to human studies without restriction of the publication language. The reference lists of original and review articles were also analyzed manually. The final literature search was performed on August 24, 2019.

### Study selection

Studies were included if they met the following criteria: (1) published as full-length article; (2) designed as cohort studies with the minimal follow-up duration of 1 year; (3) included women with breast cancer; (4) MetS was identified as exposure of interest at baseline; (5) documented the incidence of at least one of the outcomes during follow-up, including the primary outcome of breast cancer recurrence, and the secondary outcomes of breast cancer related deaths and all-cause deaths; and (6) reported the adjusted risk ratios (RRs, at least adjusted for age) and their corresponding 95% confidence intervals (CIs) for the above outcomes comparing breast cancer women with and without MetS. Definitions of MetS were consistent with that was applied in the original studies. Reviews, editorials, preclinical studies, and non-cohort studies were excluded.

### Data extracting and quality evaluation

Literature search, data extraction, and study quality assessment were independently performed by two authors according to the predefined inclusion criteria. If inconsistencies occurred, discussion with the corresponding author was suggested to resolve these issues. The following data were extracted: (1) name of the first author, publication year, study location, and study design; (2) characteristics and numbers of women with breast cancer, ethnic groups, criteria for the diagnosis of MetS, and follow-up period; and (3) number of cases with breast cancer recurrence, breast cancer related deaths, and all-cause deaths during follow-up, and variables adjusted when presenting the RRs. The quality of each study was evaluated using the Newcastle–Ottawa Scale (NOS) [27]. This scale ranges from 1 to 9 stars and judges the quality of each study regarding three aspects: selection of the study groups; the comparability of the groups; and the ascertainment of the outcome of interest.

### Statistical analyses

The association between MetS and breast cancer recurrence or mortality outcome was measured by RRs in this study. To stabilize its variance and normalize the distribution, RR data and its corresponding stand error (SE) from each study was logarithmically transformed [26]. The Cochrane’s Q test was performed to evaluate the heterogeneity among the include cohort studies [26, 28], and the I^2^ statistic was also calculated. A significant heterogeneity was considered if I^2^ > 50%. A random effect model was used to pool the results since this model has been indicated to incorporate the potential heterogeneity of the included studies and therefore could provide a more generalized result. Sensitivity analysis by omitting one study at a time was performed to evaluate the stability of the results [26]. To evaluate the influences of ethnicity and study design on the outcome, predefined subgroup analyses were performed [29]. Potential publication bias was assessed by visual inspection of the symmetry of the funnel plots, complemented with the Egger regression test [30]. The RevMan (Version 5.1; Cochrane Collaboration, Oxford, UK) and STATA software were used for the statistics.

## Results

### Literature search

**Fig. 1:**
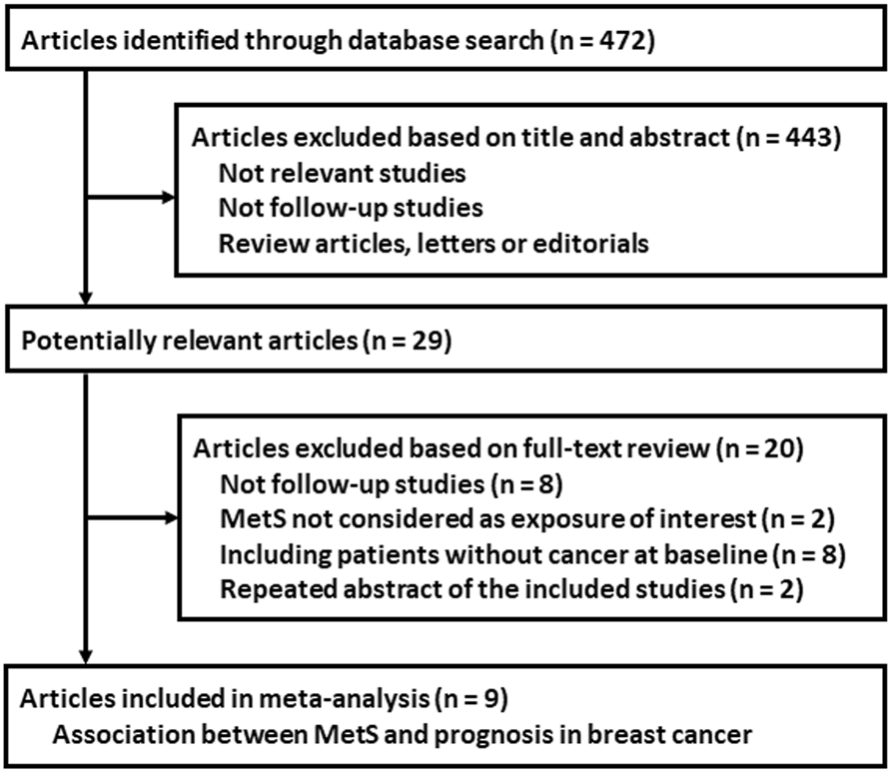
Flowchart of database search and study inclusion

The flowchart of database search was shown in Fig. 1. Briefly, 472 studies were obtained from database search, and 443 of them were excluded primarily due to the irrelevance to the objective of the study. For the remaining 29 potential relevant studies that underwent full text review, 20 were further excluded because eight of them were not cohort studies, two did not include MetS as exposure of interest, eight included patients without breast cancer at baseline, and the other two were repeated abstracts of the included studies. Finally, nine follow-up studies were included [15–23].

### Study characteristics and quality

Overall, this meta-analysis included nine cohort studies [15–23] with 17,892 women with breast cancer. Since three studies provided data based on the status of hormone receptors (HR) of the cancer [16, 19, 20] and one study based on age stratification [22], these datasets were included separately. The characteristics of the included cohorts were shown in Table 1. Three of them were prospective cohort studies [15, 18, 19], while the other six were retrospective cohort studies [16, 17, 20–23]. All of these studies included breast cancer women who received anti-cancer therapy and were with no signs of recurrence at baseline. Among them, 2583 women (14.4%) were diagnosed as MetS at baseline according to the criteria of the revised National Cholesterol Education Program’s Adults Treatment Panel III (NCEP-ATP III), the International Diabetes Federation (IDF), American Association of Clinical Endocrinologists (AACE), and American Heart Association (AHA)/National Heart, Lung, and Blood Institute (NHLBI) [31–34]. The mean follow-up durations varied from 2.4 to 11.1 years, and outcomes of breast cancer recurrence, breast cancer related deaths, and all-cause deaths were reported. Potential confounding factors, including age, cancer stage at diagnosis, HR status, and treatment were adjusted to a varying degree in the included studies. The qualities of the included follow-up studies were generally good, with the NOS ranging from seven to nine points (Table 2).

**Table 1:** Characteristics of the included cohort studies

Study	Country	Ethnicity	Design	Patient characteristics	Sample size	Mean age years	MetS diagnosis	MetS at baseline n (%)	Follow-up duration years	Outcomes reported	Outcome validation	Variables adjusted	NOS
Pasanisi [15]	Italy	Caucasians	PC	Postmenopausal BC women 1 y after surgey, not undergoing chemotherapy	110	56.8	NCEP-ATP III	16 (14.5)	5.5	Recurrence (32)	Hospital records	Age, stage at diagnosis, HR status, current tamoxifen treatment, time between diagnosis and recruitment, serum testosterone	7
Oh [19]	Korea	Asians	PC	Newly diagnosed BC women after surgery	747	45.9	AHA/NHLBI	268 (35.9)	5.2	Recurrence (94) stratified by ER/PR status	Hospital records	Age, alcohol consumption, BMI, regional lymph node metastasis, tumor size, and chemotherapy	9
Calip [17]	US	Caucasians (90%)	RC	BC women 4 m after surgery	4216	62.2	NCEP-ATP III	1011 (23.9)	6.3	Recurrence (415), BC-related death (259), and all-cause death (1096)	Hospital records	Age, stage at diagnosis, HR status, primary treatment, race, menopausal status	8
Berrino [16]	Italy	Caucasians	RC	Stage I-III BC women after surgery	2092	51.4	IDF	419 (20.0)	2.8	Recurrence (94) stratified by ER status (164)	Hospital records	Age, education, stage at diagnosis, and the HR status	8
Fan [20]	China	Asians	RC	Stage I-III BC women	1249	49	AACE	206 (16.5)	6.6	Recurrence (265) and all-cause death (242) by HR status	Hospital records	Age, stage at diagnosis, surgery model, radiotherapy, and chemotherapy regimens	8
Muniz [21]	The US	Caucasians	RC	Stage I-II BC women with HR + , HER2-	534	56	NCEP-ATP III	117 (22.0)	4.4	Recurrence (24)	Hospital records	Age and stage at diagnosis	7
Simon [18]	The US	Caucasians (94%)	PC	Postmenopausal BC women	8641	62.9	AHA/NHLBI	423 (4.9)	11.3	BC-related death (619), and all-cause death (2181)	Medical record review linkage to the National Death Index	Age, stage at diagnosis, treatment, HR status, other comorbidities	8
Grybach [22]	Ukraine	Caucasians	RC	Stage I-III BC women	202	51.2	NCEP-ATP III	94 (46.5)	5	All-cause death (44) stratified by age	Hospital records	Age, stage at diagnosis, and HR status	7
Tong [23]	China	Asians	RC	HER2-positive BC women receiving neoadjuvant therapy	101	50.9	AHA/NHLBI	29 (28.7)	2.4	Recurrence (15)	Hospital records	Age, stage at diagnosis, HR status, and treatments	7

*PC* prospective cohort, *RC* retrospective cohort, *MetS* metabolic syndrome, *NOS* the Newcastle–Ottawa Score, *BC* breast cancer, *HER*-*2* human epidermal growth factor receptor-2, *HR* hormone receptor, *ER* estrogen receptor, *PR* progesterone receptor, *NCEP*-*ATP III* National Cholesterol Education Program’s Adults Treatment Panel III, *IDF* International Diabetes Federation, *AHA* American Heart Association, *AACE* American Association of Clinical Endocrinologists, *NHLBI* National Heart, Lung, and Blood Institute, *BMI* body mass index

**Table 2:** Details of study quality evaluation via the Newcastle–Ottawa Scale

Study	Representativeness of the exposed cohort	Selection of the non-exposed cohort	Ascertainment of exposure	Outcome not present at baseline	Control for age	Control for other confounding factors	Assessment of outcome	Enough long follow-up duration	Adequacy of follow-up of cohorts	Total
Pasanisi [15]	0	1	1	1	1	1	0	1	1	7
Oh [19]	1	1	1	1	1	1	1	1	1	9
Calip [17]	0	1	1	1	1	1	1	1	1	8
Berrino [16]	1	1	1	1	1	1	1	0	1	8
Fan [20]	1	1	1	1	1	1	1	1	0	8
Muniz [21]	0	1	1	1	1	0	1	1	1	7
Simon [18]	1	1	1	1	1	1	1	1	0	8
Grybach [22]	0	1	1	1	1	1	1	1	0	7
Tong [23]	0	1	1	1	1	1	1	0	1	7

### Association between MetS and recurrence risk of breast cancer

**Fig. 2:**
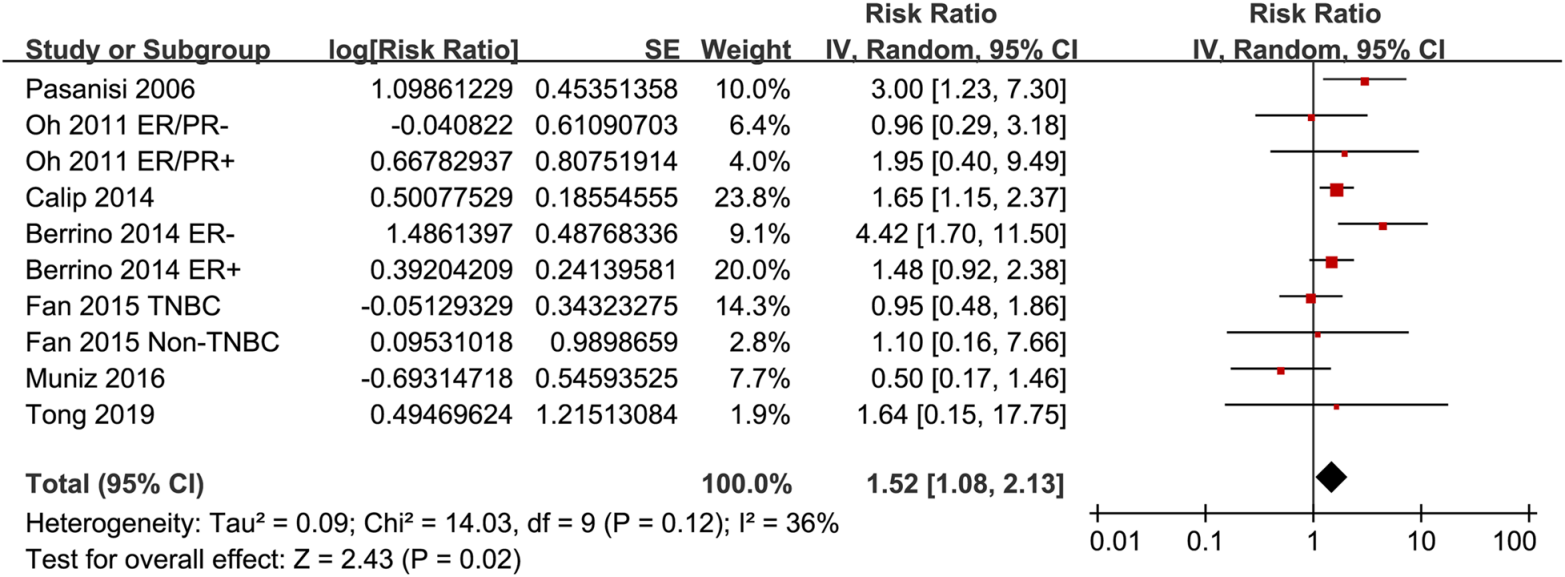
Meta-analysis for the association between MetS and recurrence of breast cancer

**Fig. 3:**
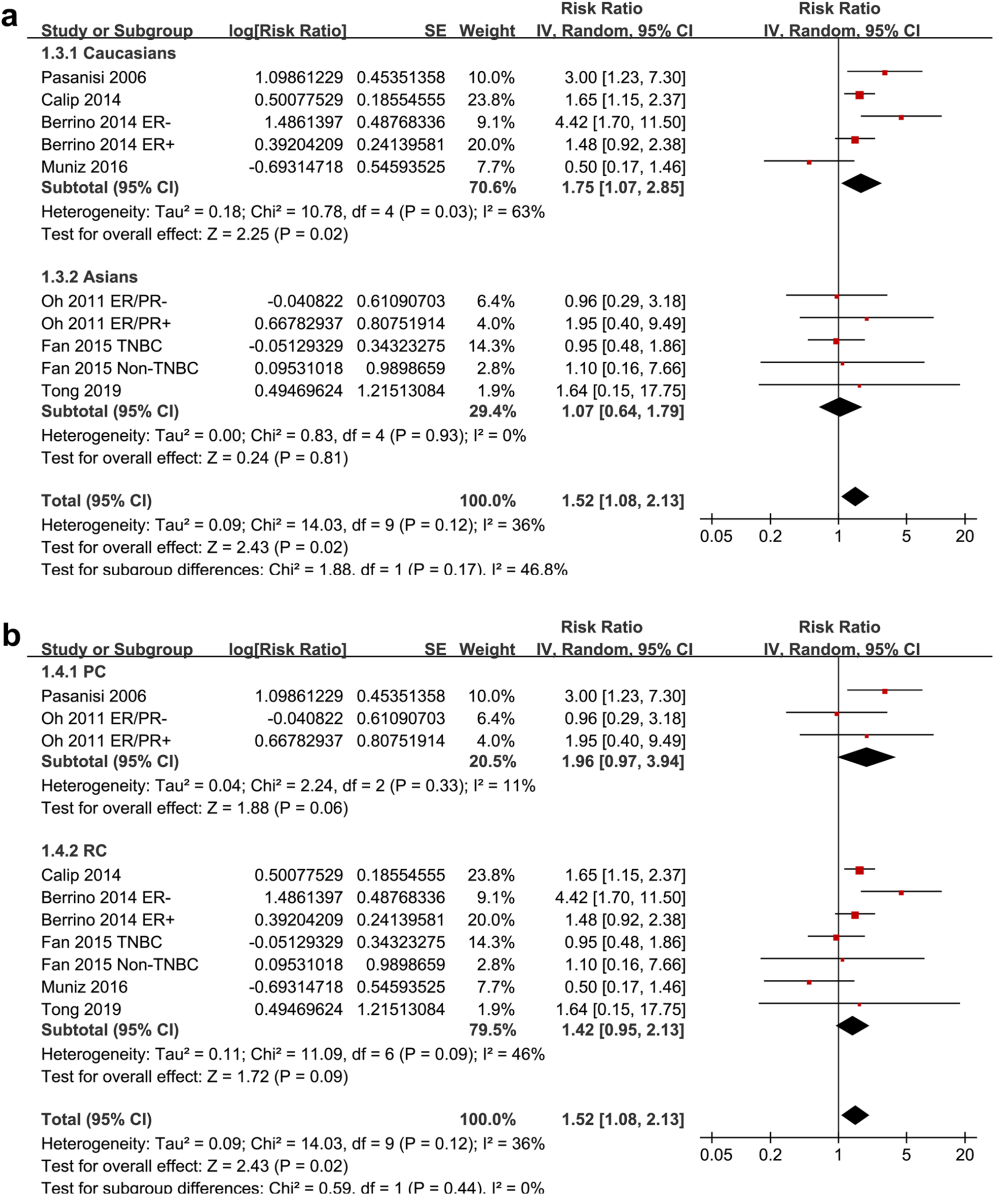
Subgroup analyses for the association between MetS and recurrence of breast cancer. **a** Stratified by ethnicity; and **b** stratified by study design

Ten datasets from seven cohort studies [15–17, 19–21, 23] were included for the meta-analysis of the association between MetS and recurrence risk in women with breast cancer. Moderate heterogeneity was detected (p for Cochrane’s Q test = 0.12, I^2^ = 36%). Pooled results with a random-effect model showed that MetS at baseline was significantly associated with increased recurrence risk of breast cancer (adjusted RR = 1.52, 95% CI 1.08 to 2.13, p = 0.02; Fig. 2). Results of sensitivity analyses by omitting one study at a time did not significantly change the results (adjusted RR: 1.44 to 1.64, p all < 0.05), suggesting the robustness of the finding. Subgroup analysis by the ethnicity of the included women showed that MetS was independently associated with increased recurrence risk of breast cancer in Caucasians (five datasets, adjusted RR = 1.75, 95% CI 1.07 to 2.85; p = 0.02), but not in Asians (five datasets, adjusted RR = 1.07, 95% CI 0.64 to 1.79; p = 0.81; Fig. 3a). Subgroup analyses according to the study design showed that MetS was associated with a trend of increased recurrence risk of breast cancer in both the prospective and retrospective cohort studies (Fig. 3b).

### Association between MetS and mortality risk in women with breast cancer

**Fig. 4:**
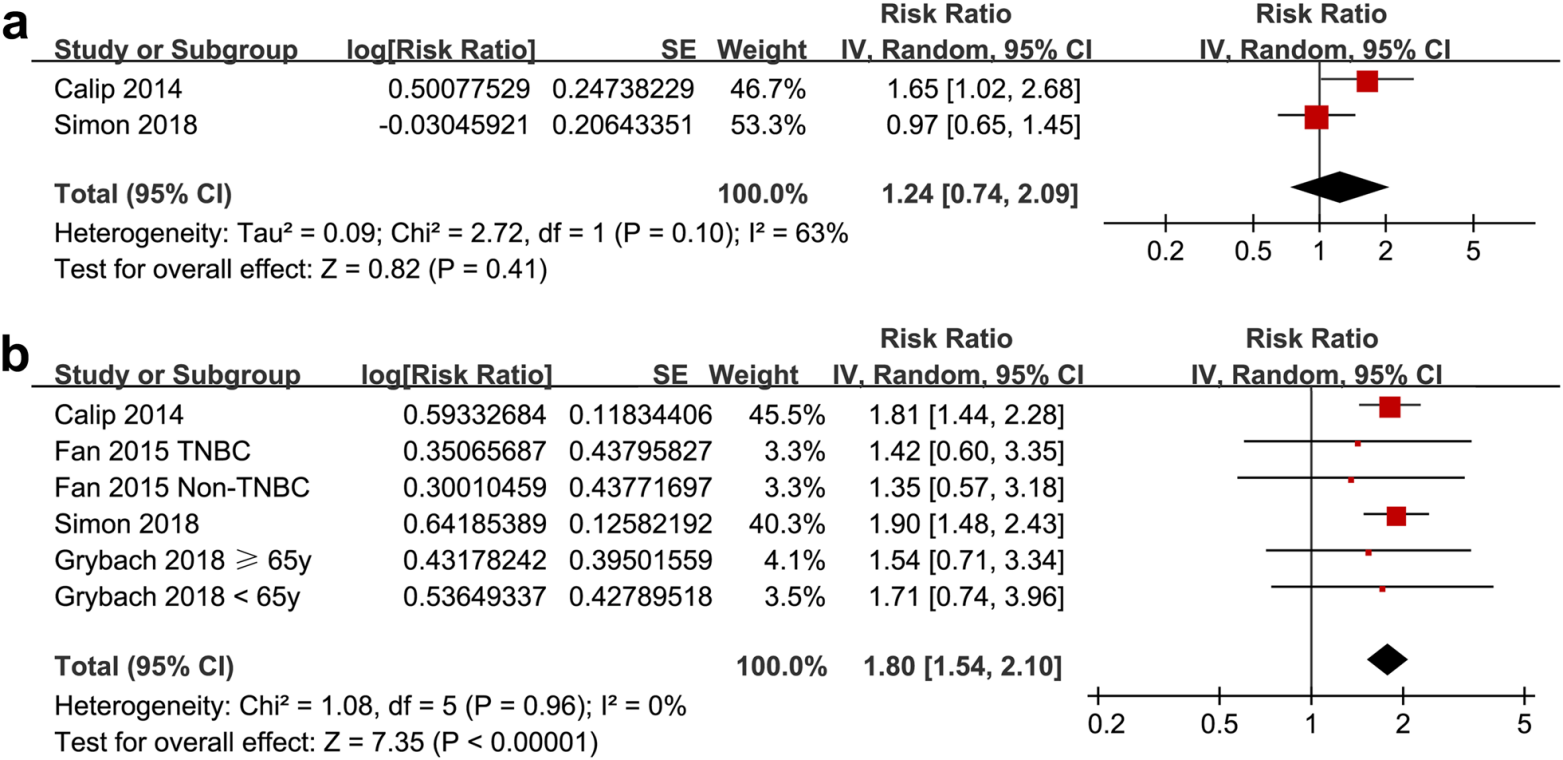
Meta-analysis for the association between MetS and mortality in women with breast cancer. **a** Breast cancer related deaths; and **b** all-cause deaths

Meta-analysis of two studies [17, 18] showed that MetS was not significantly associated with increased risk of breast cancer related deaths (adjusted RR = 1.24, 95% CI 0.74 to 2.09; p = 0.41; I^2^ = 63%; Fig. 4a). However, meta-analysis of six datasets from four studies [17, 18, 20, 22] showed that MetS was significantly associated with an increased risk of all-cause deaths in women with breast cancer (adjusted RR = 1.80, 95% CI 1.54 to 2.10; p < 0.001; I^2^ = 0%; Fig. 4b).

### Publication bias

**Fig. 5:**
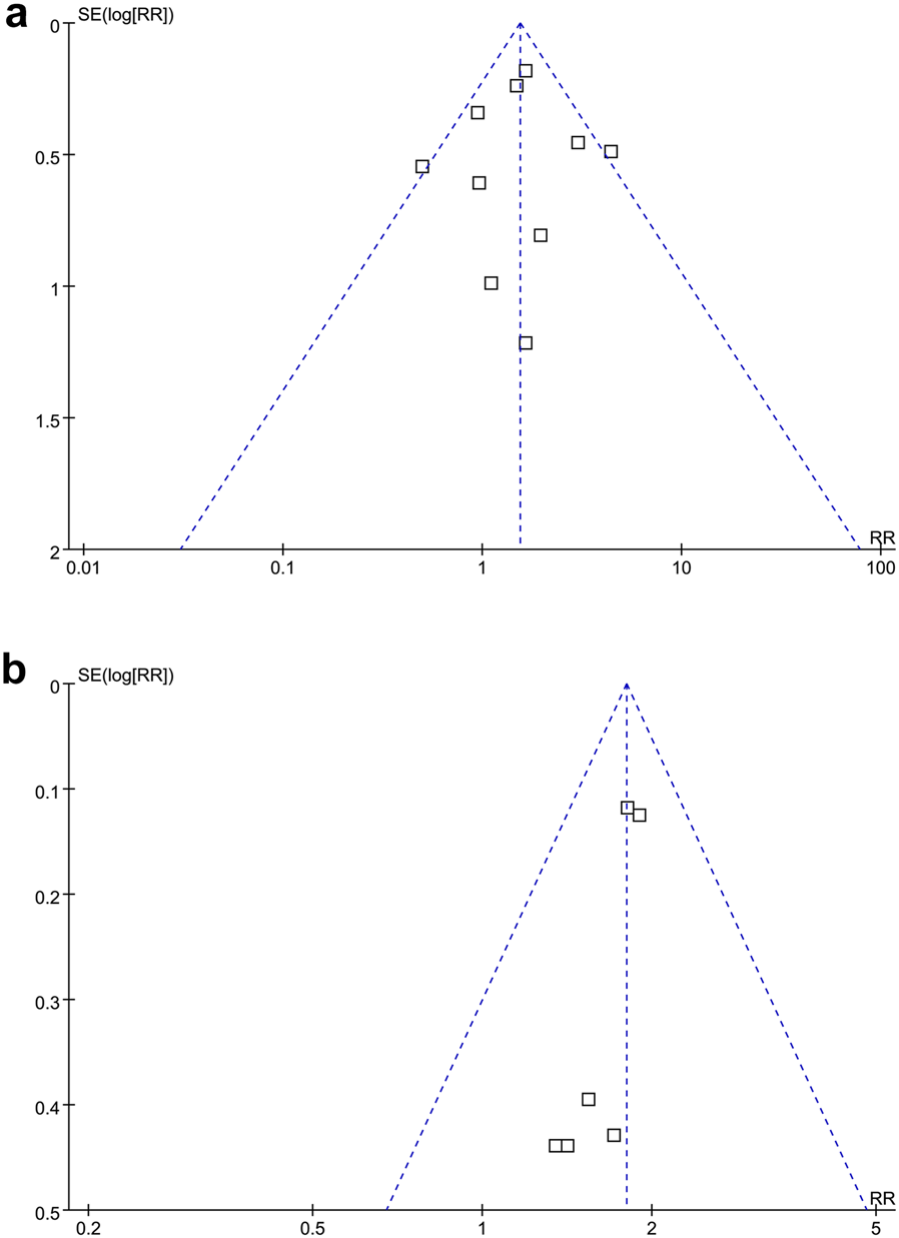
Funnel plots for the meta-analyses of the association between MetS and prognosis of breast cancer. **a** recurrence of breast cancer; and **b** all-case deaths

The funnel plots for the association between MetS and risks of recurrence and all-cause deaths in women with breast cancer were shown in Fig. 5a, b. The plots were symmetrical on visual inspection, suggesting low risks of publication biases. Results of Egger’s regression tests also showed similar results (p = 0.542 and 0.344, respectively). Publication bias for the meta-analysis of the association between MetS and risk of breast cancer related deaths was difficult to estimate since only two studies were included.

## Discussion

In this meta-analysis of longitudinal follow-up studies, we found that MetS in women with breast cancer may predict poor clinical prognosis. Specifically, MetS was associated with an increased risk of breast cancer recurrence, even after controlling of potential confounding factors including age, disease stage at diagnosis, HR status, and treatments. Moreover, we found that the association between MetS and increased recurrence risk of breast cancer was significant in studies including Caucasians, but not in Asians. In addition, although a significant association between MetS and breast cancer related deaths was not retrieved by meta-analysis of only two studies, we found that MetS was significantly associated with increased all-cause deaths in women with breast cancer. These results demonstrated that MetS may be a prognostic factor in women with breast cancer, which may predict higher risk of overall mortality.

To the best of our knowledge, our study is the first meta-analysis to evaluate the potential association between MetS and clinical outcomes in women with breast cancer. By combining the results of multivariable-adjusted data, results of our study showed that MetS in women with breast cancer is associated with higher recurrence risk and overall mortality, which is independent of some known prognostic factors, such as age, disease stages, and HR status. These results highly indicated that MetS may be an independent predictor of poor prognosis in women with breast cancer. These findings should be validated in large-scale prospective cohort studies, and clinical studies are needed to determine whether optimized management of MetS in women with breast cancer could improve their clinical outcomes. Another interesting finding of the study is that our subgroup analysis showed that MetS is associated with a higher recurrence risk of breast cancer in Caucasian women, but not in Asian women. Although the mechanisms underlying the potential racial difference between the association of MetS and prognosis in breast cancer remain undetermined, some previous studies did indicate a potential racial difference regarding the influence of comorbidities on the survival in women with breast cancer. A previous retrospective cohort study using the Surveillance, Epidemiology and End Results-Medicare linked data showed that diabetes was associated with increased breast cancer-specific mortality in white women, but not in the other ethnicities [35]. Moreover, previous studies indicated that prevalence of breast cancer subtypes varied by race/ethnicity [36, 37], which may also be the reason of the potential mechanism underlying the racial difference between the association of MetS and prognosis in women with breast cancer. Further studies investigating the potential mechanisms are warranted.

The potential pathophysiological mechanisms underlying the association between MetS and poor prognosis in breast cancer may be multifactorial. Previous clinical studies showed that MetS may be associated with more aggressive tumor biology of breast cancer [13, 14], although different findings are also shown from another study [38]. Insulin resistance and chronic inflammation are the characterized pathophysiological features in MetS [39]. Experimental studies showed that insulin resistance could lead to compensatory hyperinsulinemia, which enhanced the cross-binding of insulin to the insulin-like growth factor-1 (IGF-1) receptors expressed on breast epithelial cells [40]. The activated IGF-1 pathway may stimulate the carcinogenesis and progression of breast cancer [40]. Moreover, hyperinsulinemia may also accelerate the progression of breast cancer by stimulation of hepatic IGF-1 synthesis and inhibition the hepatic expression of IGF-1 receptors, leading to an increased circulating IGF-1 level [40]. Also, the chronic low-grade inflammation in MetS patients has also been involved in the development and aggression of many malignancies, including breast cancer [41]. A previous study in obesity-resistant BALB/c strain of female mice showed that a high-fat diet could stimulate growth of an estrogen receptor (ER) -negative murine mammary carcinoma cell line, and its metastasis from the orthotropic injection site to the lungs and liver. This accelerated cancer progression was accompanied by enhanced tumor-related angiogenesis and increased serum concentrations of several proinflammatory cytokines, including interleukin 6, and leptin, which suggested the potential association between MetS, inflammation, and cancer invasion [42]. Moreover, in women with breast cancer, inflammation in the tumor microenvironment, with local elevation in the expression of proinflammatory cytokines (such as tumor necrosis factor-α), has also been associated with increased invasiveness and a poor prognosis [43]. Although all of the components of MetS have been linked with an increased risk of breast cancer in postmenopausal women in a previous meta-analysis, the combination of these components in MetS seemed to confer stronger association than individual components [44]. The key mechanisms and the exact molecular signaling pathways underling the association between MetS and poor prognosis in women with breast cancer deserve further investigation.

Our study has limitations, which should be considered when interpreting the results. Firstly, as a meta-analysis of observational studies, although we combined RR data after adjustment of potential confounding factors, we could not exclude other residual factors that may confound the association between MetS and recurrence of breast cancer, such as treatment with metformin or making diet and or exercise in breast cancer survivors to modify the components of the MetS, which have all been suggested to confer anticancer efficacy [45–47]. Secondly, MetS were diagnosed with various criteria in the included studies. Although these criteria were based on the same components for the diagnosis of MetS [7, 31, 32], the differences of the criteria may be a source of heterogeneous of the meta-analysis. Importantly, the prevalence of MetS may be varying according to the different criteria used for the diagnosis of MetS. For example, among 168 Spanish postmenopausal women with breast cancer, the prevalence of NCEP-ATP III defined MetS was 53.7% [14], while another study showed that the prevalence of MetS defined by IDF criteria was 39% in another cohort of postmenopausal women with breast cancer [13]. However, due to the limited datasets available, we were unable to determine the potential influences of the different diagnostic criteria for MetS on the outcomes of the meta-analysis (Additional file 1: Figure S1). Thirdly, it has been suggested that the potential pathophysiological association between MetS (such as insulin resistance and inflammation) and breast cancer may be affected by hormone status of the cancer [4]. Therefore, it is of clinical importance to determine the potential influence of HR status on the association between MetS and outcomes of breast cancer. However, only three of the included studies provided data stratified by HR status of the cancer [16, 19, 20]. One focused on the status of estrogen receptor (ER) [16], one analyzed ER and progesterone receptor (PR) [19], and another one evaluated ER, HR and human epidermal growth factor receptor-2 (HER-2) [20]. The differences of the HRs analyzed in the above studies made it unable to perform a subgroup analysis with available data. Therefore, further large-scale prospective cohort studies are needed to determine the association between MetS and clinical outcomes in women with breast cancer of different HR status and of different menopausal status. Fourthly, due to the limited number of the included studies, result for the association between MetS and breast cancer related mortality should be interpreted with caution until further studies are available. Fifthly, the mean follow-up durations varied significantly across studies (from 2.4 to 11.1 years), and including very short follow-up studies may affect the outcome of the meta-analysis. In addition, menopausal status has been shown to affect the association between MetS and risk of breast cancer in a previous meta-analysis [44]. Similarly, in the Me-Can (metabolic syndrome and cancer) project, MetS was associated with a decreased risk of incident breast cancer in women below age 50 with high body mass index, and with an increased risk of breast cancer mortality in women above 60 [48]. It remains unknown whether menopausal status may affect the association between MetS and prognosis in women with breast cancer, and further researches are needed. Finally, a causative relationship between MetS and poor prognosis in breast cancer should not be retrieved from our results. Future studies are needed to determine whether management of MetS in women with breast cancer could improve their clinical outcomes.

## Conclusions

In conclusion, our meta-analysis showed that MetS may predict higher risk of cancer recurrence and mortality in women with breast cancer, particularly in Caucasians. Future studies are needed to clarify the potential influence of cancer HR status and menopausal status on the association between MetS and prognosis, and to determine whether management of MetS in women with breast cancer could improve their clinical outcomes.

## Supplementary information

**Additional file 1: Figure S1.** Subgroup analyses for the association between MetS and recurrence of breast cancer according to the diagnostic criteria of MetS.

## Data Availability

The available data and materials section refers to the raw data used in our study are included in manuscript with tables, figures and its additional files. All the authors agreed that the data could be shared if researchers required.

## Abbreviations

MetS: metabolic syndrome
GC: gastric cancer
MOOSE: Meta-analysis of Observational Studies in Epidemiology
RRs: risk ratios
SE: stand error
NCEP-ATP III: National Cholesterol Education Program’s Adults Treatment Panel III
IDF: International Diabetes Federation
HR: hormone receptors
IGF: insulin-like growth factor-1
ER: estrogen receptor
PR: progesterone receptor
HER-2: human epidermal growth factor receptor-2
