# Supplementary material for: Association between metabolic syndrome and prognosis of breast cancer: a meta-analysis of follow-up studies
Source: Diabetol Metab Syndr. 2020 Jan 29;12:10. doi: 10.1186/s13098-019-0514-y (PMC6990514; doi:10.1186/s13098-019-0514-y)

**Figure S1** Subgroup analyses for the association between MetS and recurrence of breast cancer according to the diagnostic criteria of MetS


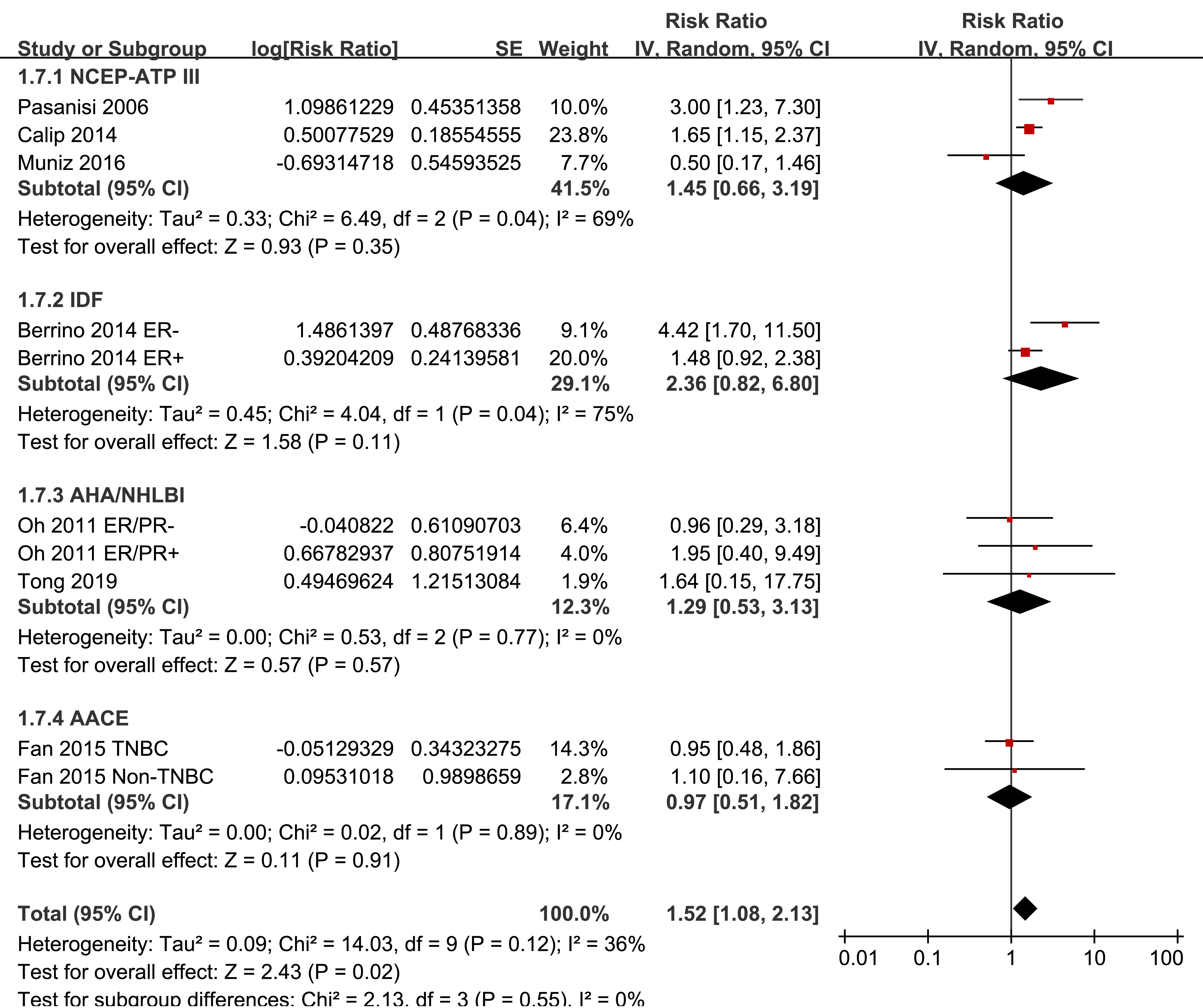

Supplement: Supplementary file 1 — Additional file 1: Figure S1. Subgroup analyses for the association between MetS and recurrence of breast cancer according to the diagnostic criteria of MetS. [file 13098_2019_514_MOESM1_ESM.docx]
